# Supplementary material for: An open-source framework for large-scale, flexible evaluation of biomedical text mining systems
Source: J Biomed Discov Collab. 2008 Jan 29;3:1. doi: 10.1186/1747-5333-3-1 (PMC2276192; doi:10.1186/1747-5333-3-1)
Supplement: Additional file 1 — Evaluation platform source code. The additional file contains the software evaluation framework discussed in this paper. The most current release can be downloaded from SourceForge.net [1]. See the accompanying README file for instructions on installation and use. Also included in the distribution is a collection of UIMA wrappers for some commonly used BioNLP tools and annotated corpora. [file 1747-5333-3-1-S1.zip › bionlp_uima_repository-1.0.1/docs/api/src-html/edu/uchsc/ccp/util/nlp/mention/ClassMention.html]

```
001    /*
002     *
003     * The Original Code is UCHSC Utilities.
004     *
005     * The Initial Developer of the Original Code is University of Colorado.  
006     * Copyright (C) 2005.  All Rights Reserved.
007     *
008     * UCHSC Utilities was developed by the Center for Computational Pharmacology
009     * (http://compbio.uchsc.edu) at the University of Colorado Health 
010     *  Sciences Center School of Medicine with support from the National 
011     *  Library of Medicine.  
012     *
013     *
014     * Contributor(s):
015     *   William A. Baumgartner <william.baumgartner@uchsc.edu> (Original Author)
016     *   
017     * Created: November 2005
018     * Dependencies:
019     * TODO:    the getSlotMentionByName() method should return a list in case there are more than one slot by the same name.. same goes for getComplexSlotMentionByName()
020     *          The two collections (ComplexSlotMentions, and SlotMentions) could be combined into a single Mention Collection for more elegant code.
021     * Changes:
022     * 04/30/06 - wab - added getSingleLineRepresentation()
023     * 06/07/06 - wab - added equals() - this should be further updated in the future.
024     */
025    
026    package edu.uchsc.ccp.util.nlp.mention;
027    
028    import java.io.PrintStream;
029    import java.util.ArrayList;
030    import java.util.Collections;
031    import java.util.List;
032    
033    import edu.uchsc.ccp.util.nlp.annotation.TextAnnotation;
034    import edu.uchsc.ccp.util.nlp.mention.comparison.IdenticalClassMentionComparator;
035    
036    public class ClassMention extends Mention {
037        private List<SlotMention> slotMentions;
038    
039        private List<ComplexSlotMention> complexSlotMentions;
040    
041        private List<TextAnnotation> textAnnotations;
042    
043        @Deprecated
044        public ClassMention() {
045            super();
046            // initialize the mention collections
047            slotMentions = new ArrayList<SlotMention>();
048            complexSlotMentions = new ArrayList<ComplexSlotMention>();
049            textAnnotations = new ArrayList<TextAnnotation>();
050    
051        }
052    
053        public ClassMention(String mentionName) {
054            super(mentionName);
055            // initialize the mention collections
056            slotMentions = new ArrayList<SlotMention>();
057            complexSlotMentions = new ArrayList<ComplexSlotMention>();
058            textAnnotations = new ArrayList<TextAnnotation>();
059    
060        }
061    
062        public List<TextAnnotation> getTextAnnotations() {
063            return textAnnotations;
064        }
065    
066        public void setTextAnnotation(List<TextAnnotation> textAnnotations) {
067            this.textAnnotations = textAnnotations;
068        }
069    
070        public void addTextAnnotation(TextAnnotation textAnnotation) {
071            textAnnotations.add(textAnnotation);
072        }
073    
074        public List<ComplexSlotMention> getComplexSlotMentions() {
075            return complexSlotMentions;
076        }
077    
078        public void setComplexSlotMentions(List<ComplexSlotMention> complexSlotMentions) {
079            this.complexSlotMentions = complexSlotMentions;
080        }
081    
082        public List<SlotMention> getSlotMentions() {
083            return slotMentions;
084        }
085    
086        public void setSlotMentions(List<SlotMention> slotMentions) {
087            this.slotMentions = slotMentions;
088        }
089    
090        public void addComplexSlotMention(ComplexSlotMention complexSlotMention) {
091            this.complexSlotMentions.add(complexSlotMention);
092        }
093    
094        public void addSlotMention(SlotMention slotMention) {
095            this.slotMentions.add(slotMention);
096        }
097    
098        /**
099         * Returns the unique names of all slotmentions
100         * 
101         * @return
102         */
103        public List<String> getSlotMentionNames() {
104            List<String> slotMentionNames = new ArrayList<String>();
105    
106            for (SlotMention sm : this.slotMentions) {
107                String name = sm.getMentionName();
108                if (!slotMentionNames.contains(name)) {
109                    slotMentionNames.add(name);
110                }
111            }
112    
113            return slotMentionNames;
114        }
115    
116        @Deprecated
117        public SlotMention getSlotMentionByName(String slotMentionName) {
118            for (SlotMention sm : this.getSlotMentions()) {
119                if (sm.getMentionName().equalsIgnoreCase(slotMentionName)) {
120                    return sm;
121                }
122            }
123            /* if we didn't find a slot by that name, create one and return it */
124            SlotMention sm = new SlotMention(slotMentionName);
125            this.addSlotMention(sm);
126            return sm;
127        }
128    
129        /**
130         * Return all slot mentions for a given name. If there are no slot mentions for a given name, null is returned.
131         * 
132         * @param slotMentionName
133         * @return
134         */
135        public List<SlotMention> getSlotMentionsByName(String slotMentionName) {
136            List<SlotMention> slotMentions = new ArrayList<SlotMention>();
137            for (SlotMention sm : this.getSlotMentions()) {
138                if (sm.getMentionName().equalsIgnoreCase(slotMentionName)) {
139                    slotMentions.add(sm);
140                }
141            }
142            if (slotMentions.size() > 0) {
143                return slotMentions;
144            } else {
145                return null;
146            }
147        }
148    
149        @Deprecated
150        public ComplexSlotMention getComplexSlotMentionByName(String complexSlotMentionName) {
151            for (ComplexSlotMention csm : this.getComplexSlotMentions()) {
152                if (csm.getMentionName().equalsIgnoreCase(complexSlotMentionName)) {
153                    return csm;
154                }
155            }
156            /* if we didn't find a slot by that name, create one and return it. */
157            ComplexSlotMention csm = new ComplexSlotMention(complexSlotMentionName);
158            this.addComplexSlotMention(csm);
159            return csm;
160        }
161    
162        /**
163         * Return all complex slot mentions for a given name. If there are no complex slot mentions for a given name, null is returned.
164         * 
165         * @param complexSlotMentionName
166         * @return
167         */
168        public List<ComplexSlotMention> getComplexSlotMentionsByName(String complexSlotMentionName) {
169            List<ComplexSlotMention> csmList = new ArrayList<ComplexSlotMention>();
170            for (ComplexSlotMention csm : this.getComplexSlotMentions()) {
171                if (csm.getMentionName().equalsIgnoreCase(complexSlotMentionName)) {
172                    csmList.add(csm);
173                }
174            }
175            if (csmList.size() > 0) {
176                return csmList;
177            } else {
178                return null;
179            }
180            // /* if we didn't find a slot by that name, create one and return it. */
181            // ComplexSlotMention csm = new ComplexSlotMention(complexSlotMentionName);
182            // this.addComplexSlotMention(csm);
183            // return csm;
184        }
185    
186        /**
187         * Return the first slot mention
188         * 
189         * @return
190         */
191        public SlotMention getFirstSlotMention() {
192            for (SlotMention sm : this.getSlotMentions()) {
193                return sm;
194            }
195            return null;
196        }
197    
198        /**
199         * outputs a single line representation of this class mention. Slots are alphabetized before output so that they are always in the same order
200         * 
201         * @return
202         */
203        public String getSingleLineRepresentation() {
204            TextAnnotation ta = getTextAnnotations().get(0);
205            String repStr = " [" + getMentionName() + "]" + ta.getSingleLineRepresentation(true, false) + "(";
206    
207            Collections.sort(getComplexSlotMentions(), new MentionSortComparator());
208            Collections.sort(getSlotMentions(), new MentionSortComparator());
209    
210            for (ComplexSlotMention csm : getComplexSlotMentions()) {
211                repStr += csm.getSingleLineRepresentation();
212            }
213            for (SlotMention sm : getSlotMentions()) {
214                repStr += sm.getSingleLineRepresentation();
215            }
216    
217            repStr += ")";
218            return repStr;
219        }
220        
221        /**
222         * similar to getSingleLineRepresentation, however this is a document-level representation, so no meta data are included (span info, covered text, etc.)
223         * 
224         * @return
225         */
226        public String getDocumentLevelSingleLineRepresentation() {
227            TextAnnotation ta = getTextAnnotations().get(0);
228            String repStr = " [" + getMentionName() + "]" + ta.getDocumentLevelSingleLineRepresentation(false) + "(";
229    
230            Collections.sort(getComplexSlotMentions(), new MentionDocumentLevelSortComparator());
231            Collections.sort(getSlotMentions(), new MentionDocumentLevelSortComparator());
232    
233            for (ComplexSlotMention csm : getComplexSlotMentions()) {
234                repStr += csm.getDocumentLevelSingleLineRepresentation();
235            }
236            for (SlotMention sm : getSlotMentions()) {
237                repStr += sm.getDocumentLevelSingleLineRepresentation();
238            }
239    
240            repStr += ")";
241            return repStr;
242        }
243    
244        public static void printMention(ClassMention cm, PrintStream ps, int indentLevel) {
245            String indentSpace = "-";
246            for (int i = 0; i < indentLevel * 4; i++) {
247                indentSpace += " ";
248            }
249            if (cm != null) {
250                String annotationString = "";
251                if (cm.getTextAnnotations().size() > 0) {
252                    annotationString = cm.getTextAnnotations().get(0).getCoveredText();
253                }
254                ps.println(indentSpace + "CLASS MENTION: " + cm.getMentionName() + " \"" + annotationString + "\"");
255                indentLevel++;
256                for (SlotMention sm : cm.getSlotMentions()) {
257                    SlotMention.printMention(sm, ps, indentLevel);
258                }
259                for (ComplexSlotMention csm : cm.getComplexSlotMentions()) {
260                    ComplexSlotMention.printMention(csm, ps, indentLevel);
261                }
262    
263            } else {
264                ps.println(indentSpace + "CLASS MENTION: null");
265            }
266    
267        }
268    
269        public String getHashKey() {
270    
271            // String key = this.getMentionName();
272            // for (ComplexSlotMention csm : this.getComplexSlotMentions()) {
273            // key += (" " + csm.getHashKey());
274            // }
275            // for (SlotMention sm : this.getSlotMentions()) {
276            // key += (" " + sm.getHashKey());
277            // }
278            // return key;
279    
280            String hashKey = "";
281            try {
282                hashKey = textAnnotations.get(0).getHashKey();
283            } catch (Exception e) {
284                System.err
285                        .println("ClassMention is missing referential TextAnnotation, therefore proper hashkey cannot be produced... ClassMention: ");
286                printMention(this, System.out, 0);
287                e.printStackTrace();
288            }
289    
290            return hashKey;
291        }
292    
293        /**
294         * The default compareTo uses the IdenticalClassMentionComparator()
295         */
296        public int compareTo(Object o) {
297            if (o instanceof ClassMention) {
298                ClassMention cmToCompare = (ClassMention) o;
299                IdenticalClassMentionComparator icmc = new IdenticalClassMentionComparator();
300                return icmc.compare(this, cmToCompare);
301            } else {
302                System.err.println("Unexpected object when comparing to ClassMention: object = " + o.getClass().getName());
303                return -1;
304            }
305    
306        }
307    
308        /**
309         * The default equals() method uses the IdenticalClassMentionComparator.
310         */
311        @Override
312        public boolean equals(Object classMentionToEquate) {
313            if (!(classMentionToEquate instanceof ClassMention)) {
314                throw new ClassCastException("A ClassMention object expected.");
315            } else {
316                ClassMention cm = (ClassMention) classMentionToEquate;
317    
318                IdenticalClassMentionComparator icmc = new IdenticalClassMentionComparator();
319                
320                if (icmc.compare(this, cm) == 0) {
321                    return true;
322                } else {
323                    return false;
324                }
325            }
326        }
327    
328    //    /**
329    //     * Check for equality among two class mentions, however this method provides an option for checking the linked TextAnnotation. If true, then the
330    //     * equals() method is used. If false, the class mention name and slots are compared, however the text annotations linked to the class mentions
331    //     * being compared are assumed to be equal. This flag enables the recursive comparison of two text annotations by avoiding an infinite loop where
332    //     * the text annotations are compared, then the class mentions are compared, and then the text annotations are compared again, etc.
333    //     * 
334    //     * @param cm
335    //     * @param checkTextAnnotations
336    //     * @return
337    //     */
338    //    public boolean equals(ClassMention cm, boolean compareTextAnnotations) {
339    //        if (compareTextAnnotations) {
340    //            return this.equals(cm);
341    //        } else {
342    //            if (cm.getMentionName().equals(this.getMentionName())) {
343    //                boolean slotMentionsMatch = compareSlotMentions(cm.getSlotMentions());
344    //                boolean complexSlotMentionsMatch = compareComplexSlotMentions(cm.getComplexSlotMentions());
345    //                return slotMentionsMatch && complexSlotMentionsMatch;
346    //            } else {
347    //                return false;
348    //            }
349    //        }
350    //    }
351    
352        // public int compareTo(Object o) {
353        // if (o instanceof ClassMention) {
354        // ClassMention compareCM = (ClassMention) o;
355        // return getSingleLineRepresentation().compareTo(compareCM.getSingleLineRepresentation());
356        // } else {
357        // System.err.println("Unexpected object when comparing to ClassMention: object = " + o.getClass().getName());
358        // return -1;
359        // }
360        //
361        // }
362        //
363        // /**
364        // * NOTE: ClassMentions are currently considered equivalent if their mention names are identical. In the future, slots should be checked as well.
365        // */
366        // @Override
367        // public boolean equals(Object classMentionToEquate) {
368        // if (!(classMentionToEquate instanceof ClassMention)) {
369        // throw new ClassCastException("A ClassMention object expected.");
370        // } else {
371        // ClassMention cm = (ClassMention) classMentionToEquate;
372        //
373        // if (cm.getMentionName().equals(this.getMentionName())) {
374        // /*
375        // * grab the text annotation that corresponds to this class mention and see if it is equal to the text annotation associated with the
376        // * input class mention
377        // */
378        // boolean textAnnotationsMatch = compareTextAnnotations(cm.getTextAnnotations());
379        // boolean slotMentionsMatch = compareSlotMentions(cm.getSlotMentions());
380        // boolean complexSlotMentionsMatch = compareComplexSlotMentions(cm.getComplexSlotMentions());
381        // return textAnnotationsMatch & slotMentionsMatch & complexSlotMentionsMatch;
382        // } else {
383        // return false;
384        // }
385        // }
386        // }
387        //
388        // /**
389        // * Check for equality among two class mentions, however this method provides an option for checking the linked TextAnnotation. If true, then the
390        // * equals() method is used. If false, the class mention name and slots are compared, however the text annotations linked to the
391        // * class mentions being compared are assumed to be equal. This flag enables the recursive comparison of two text annotations by avoiding an
392        // * infinite loop where the text annotations are compared, then the class mentions are compared, and then the text annotations are compared
393        // again,
394        // * etc.
395        // *
396        // * @param cm
397        // * @param checkTextAnnotations
398        // * @return
399        // */
400        // public boolean equals(ClassMention cm, boolean compareTextAnnotations) {
401        // if (compareTextAnnotations) {
402        // return this.equals(cm);
403        // } else {
404        // if (cm.getMentionName().equals(this.getMentionName())) {
405        // boolean slotMentionsMatch = compareSlotMentions(cm.getSlotMentions());
406        // boolean complexSlotMentionsMatch = compareComplexSlotMentions(cm.getComplexSlotMentions());
407        // return slotMentionsMatch && complexSlotMentionsMatch;
408        // } else {
409        // return false;
410        // }
411        // }
412        // }
413        //    
414        // /**
415        // * This method enables the user to compare class mentions/annotations to a given depth.<br>
416        // * Depth &lt;0: compare at all depths (i.e. simply use the equals() method)
417        // * Depth 0: Annotation matches, plus the top level class mention name
418        // * Depth 1: Annotation matches, plus the top level class mention name, plus the slot values of that class mention, plus complex slot mention
419        // names?
420        // * Depth 2: Class mention names in complex slot mentions match
421        // * Depth 3: Slots of class mentions in complex slot mentions match
422        // * Depth 4: Repeat step at Depth #2
423        // * @param cm
424        // * @param compareTextAnnotations
425        // * @param maxDepth
426        // * @param currentDepth
427        // * @return
428        // */
429        // public boolean equals(ClassMention cm, boolean compareTextAnnotations, int maxDepth, int currentDepth) {
430        //        
431        // }
432    
433        // /*
434        // * This method compares the linked TextAnnotation objects with those of another ClassMention. We assume, however, that there will be only one
435        // * linked TextAnnotation. This method does not deal with other cases, as they are not known to exist.
436        // */
437        // private boolean compareTextAnnotations(List<TextAnnotation> taListToCompare) {
438        // if (this.getTextAnnotations().size() != taListToCompare.size()) {
439        // return false;
440        // } else if (this.getTextAnnotations().size() != 1) {
441        // throw new UnsupportedOperationException(
442        // "Comparison of class mentions with multiple text annotations is not supported at this time. # linked text annotations: " +
443        // this.getTextAnnotations().size() + ", " + taListToCompare.size() + " -- " + this.getSingleLineRepresentation());
444        // } else {
445        // TextAnnotation ta = this.getTextAnnotations().get(0);
446        // TextAnnotation taToCompare = taListToCompare.get(0);
447        // /* check the text annotation, but do not check the class mentions again (hence the "false") */
448        // return ta.equals(taToCompare, false);
449        // }
450        // }
451        //
452        // /*
453        // * This method compares two lists of slot mentions by iterating through them. A quick check of the number of slot mentions in each list is made
454        // at
455        // * first to see if the individual comparisons can be avoided.
456        // */
457        // private boolean compareSlotMentions(List<SlotMention> smList) {
458        // if (this.getSlotMentions().size() != smList.size()) {
459        // /* A quick check of the count of slot mentions */
460        // return false;
461        // } else {
462        // /*
463        // * iterate through each slot mention and see if it has a match in the other list. We must iterate because it is possible that there could
464        // * be two slot mentions with identical names. This is not ideal, but it is possible
465        // */
466        // for (SlotMention sm : this.getSlotMentions()) {
467        // boolean slotMentionHasAMatch = false;
468        // for (SlotMention smToCompare : smList) {
469        // if (sm.equals(smToCompare)) {
470        // slotMentionHasAMatch = true;
471        // }
472        // }
473        // if (!slotMentionHasAMatch) {
474        // return false;
475        // }
476        // }
477        // /* if we have made it this far, then all slot mentions have a match */
478        // return true;
479        // }
480        // }
481        //
482        // /* This method compares two lists of complex slot mentions by iterating through them. */
483        // private boolean compareComplexSlotMentions(List<ComplexSlotMention> csmList) {
484        // if (this.getComplexSlotMentions().size() != csmList.size()) {
485        // /* a quick check of the count of the complex slot mentions */
486        // return false;
487        // } else {
488        // /* iterate through each CSM and see if it has a match */
489        // for (ComplexSlotMention csm : this.getComplexSlotMentions()) {
490        // boolean csmHashAMatch = false;
491        // for (ComplexSlotMention csmToCompare : csmList) {
492        // if (csm.equals(csmToCompare)) {
493        // csmHashAMatch = true;
494        // }
495        // }
496        // if (!csmHashAMatch) {
497        // return false;
498        // }
499        // }
500        // /* if we have made it this far, then all complex slot mentions have a match */
501        // return true;
502        // }
503        //        
504        // }
505    
506    }
```
